# An open-label, acute clinical trial in adults to assess ketone levels, gastrointestinal tolerability, and sleepiness following consumption of (*R*)-1,3-butanediol (Avela™)

**DOI:** 10.3389/fphys.2023.1195702

**Published:** 2023-06-28

**Authors:** James Lowder, Shafagh Fallah, Carolina Venditti, Kathy Musa-Veloso, Vassili Kotlov

**Affiliations:** ^1^ Principal Investigator, Impact Science Alliance, San Diego, CA, United States; ^2^ Intertek Health Sciences Inc., Mississauga, ON, Canada; ^3^ Study Coordinator, Impact Science Alliance, San Diego, CA, United States

**Keywords:** butanediol, beta-hydroxybutyrate, ketosis, ketones, BHB

## Abstract

**Introduction:** A study was undertaken to determine the acute effects of a beverage made with Avela™ (*R*)-1,3-butanediol, on blood beta-hydroxybutyrate (BHB) levels (using the Keto-Mojo monitor), gastrointestinal (GI) tolerability (using the modified visual analogue scale GI Symptoms Tool), and sleepiness (using the Stanford Sleepiness Scale).

**Methods:** Following a 12-h overnight fast, 26 healthy adults consumed one beverage containing 11.5 g of (*R*)-1,3-butanediol at each of 0, 30, and 60 min, culminating in a total intake of 34.5 g of (*R*)-1,3-butanediol. Blood BHB levels, GI tolerability, and sleepiness were assessed at baseline (0 min), and at 30, 60, 90, 120, 180, 240, and 300 min. At 240 min, a protein bar was consumed.

**Results:** The mean (±SD) BHB fasting baseline level, maximal concentration, time at maximal concentration, and incremental area under the curve over 300 min were 0.23 ± 0.21 mmol/L, 2.10 ± 0.97 mmol/L, 133.85 ± 57.07 min, and 376.73 ± 156.76 mmol/L*min, respectively. BHB levels at each time point were significantly increased relative to baseline. In females, BHB T_max_ was significantly greater (*p* = 0.046), and BHB iAUC_0–300 min_ nearly significantly greater (*p* = 0.06) than in males.

**Discussion:** The beverage formulated with Avela™ had no impact on sleepiness and was generally well-tolerated, with no or mild GI symptoms reported in most participants. Mild headaches were reported as an adverse event by five participants and judged possibly related to the study product in two of the participants.

## 1 Introduction

Ketogenesis is a process whereby the liver utilizes fatty acids to synthesize ketones: beta-hydroxybutyrate (BHB), acetoacetate, and, to a lesser degree, acetone. Ketones are produced in low amounts when dietary carbohydrates are limited (e.g., prolonged fasting or starvation) or during prolonged exercise, but they can also be produced in response to diets that are ketogenic. Under normal physiological conditions, blood BHB levels are <0.5 mmol/L (reviewed in Laffel, 1999) and, after weeks of fasting, blood BHB levels of 4–7 mmol/L have been reported (reviewed in Cahill, 2006). Previously reported ranges for blood BHB levels after exogenous ketone administration are in the range of 0.5–3.0 mmol/L (Kolb et al., 2021) or up to 7.0 mmol/L (Cox et al., 2016).

For over a century, ketogenic diets have been used as effective treatments for childhood seizures and epilepsy (Thiele, 2003). Prior to the discovery of insulin, a ketogenic diet was used as a therapeutic approach to control type 1 and 2 diabetes (Ludwig, 2020). Recently, the role of ketone bodies (synonymous with ketones) in producing energy (i.e., adenosine triphosphate) has gained traction for its potential application in the area of sports nutrition -- to improve endurance performance and recovery from exercise. Benefits in exercise performance with the consumption of ketones have been reported in some studies, while in other studies, impairments in exercise performance were reported (Cox et al., 2016; Wroble et al., 2019; Mansor and Woo, 2021; Whitfield et el., 2021; Evans et al., 2022). In a recent systematic review and meta-analysis, neither ketone esters nor ketone salts were found to improve physical performance or metabolic, respiratory, or cardiovascular responses to exercise (Valenzuela et al., 2020). Additional studies are needed to better understand the impacts of various factors (e.g., training level of athletes; type of exercise; nature, dose, and duration of ketone supplementation; and need for metabolic adaptation) on physical performance.

Ketone drinks and supplements contain exogenous sources of ketones that, following consumption, have been used to increase blood ketone concentrations to levels of ketosis (blood BHB >0.5 mmol/L) without the dietary restrictions imposed by a ketogenic diet (Stubbs et al., 2017). Examples of these exogenous ketone sources include ketone salts (KS) and BHB ketone monoesters (KME), the latter which are broken down to butanediol and BHB (Soto-Mota et al., 2020). BHB provided by ketone drinks and supplements is thought to be an energy source that could improve sporting performance and recovery, and may have implications for other endpoints such as glucose control (Falkenhain et al., 2022), immune modulation (Neudorf et al., 2019), cardiac function (Cuenoud et al., 2020; Yurista et al., 2021) and cognitive function (Poff et al., 2021). To maximize translational potential of exogenous ketone interventions, they must be well tolerated under the intended conditions of use. Studies to date of various exogenous ketone supplements have highlighted the potential for gastrointestinal (GI) or systemic symptoms to occur following their consumption. It has previously been reported that the consumption of KME can lead to mild flatulence, nausea, and dizziness when consumed at rest, and upper abdominal discomfort when consumed during exercise (Clarke et al., 2012; Egan and D’Agostino, 2016; Vandoorne et al., 2017); as well, consumption of KS drinks pre-exercise has been associated with diarrhea and vomiting (Waldman et al., 2018). Each of these studies assessing GI symptomology utilized a simplistic questionnaire (symptom/no symptom). Severity of symptoms was assessed by Stubbs et al. (2019), wherein the GI tolerability of KME and KS was reported. Severe symptoms included nausea, abdominal cramps and diarrhea following consumption of a high-dose of KS (3.2 mmol/kg body weight) in a fasted state. Symptoms resolved completely by the end of the study. KS may be associated with more severe GI symptoms than KME due to the high intakes of sodium and potassium, which can result in water retention and diarrhea secondary to a hyperosmolar gut lumen (reviewed in Stubbs et al., 2019).

1,3-butanediol, an organic compound that can be converted to BHB, has also been studied for its potential to increase ketone levels. 1,3-butanediol is converted to BHB, primarily in the liver, by alcohol and aldehyde dehydrogenases (Stubbs et al., 2021). There are two distinct stereoisomers of 1,3-butanediol: the *R* form and the *S* form; the *R* stereoisomer is naturally occurring and appears to be the configuration that can be converted to physiological ketone bodies most proficiently (Desrochers et al., 1992). To date, five clinical studies in which the efficacy and/or safety/tolerability of (*R, S*)-1,3-butanediol were assessed have been conducted (Kies et al., 1973; Tobin et al., 1975 [Studies 1 and 2]; Scott et al., 2019; Shaw et al., 2019). There were no significant adverse events (AEs) in the studies following the consumption of a beverage providing 15–40 g per day of (*R,S*)-1,3-butanediol. In the study by Shaw et al. (2019), a beverage providing 0.5 or 0.7 g/kg body weight (35–49 g for a 70-kg person) of (*R,S*)-1,3-butanediol resulted in nausea, euphoria, and dizziness; however, these adverse outcomes were mitigated with the administration of divided servings (as opposed to a single intake). Falkenhain et al. (2023) recently published the results of a clinical study in which the *R* form of 1,3-butanediol was administered to 12 healthy male and female adults. In this study, the administration of a single serving of 10 g of (*R*)-1,3-butanediol resulted in a peak blood BHB level of 1.2 mmol/L at 39 min, and an incremental area under the curve (iAUC) of 118 mmol/L x 240 min; furthermore, the serving was well-tolerated.

The primary objective of our study was to determine the level of ketones (measured as blood BHB) following the single-day consumption of 34.5 g of (*R*)-1,3-butanediol in a beverage. The secondary objectives were to assess GI symptomology, as well as any effects on sleepiness.

## 2 Materials and methods

This study was conducted in accordance with the Declaration of Helsinki, and approved by the Institutional Review Board of PearlIRB (protocol code #22-GMAT-101; Approved 12 May 2022). The study was registered at clinicaltrials.gov (NCT05384106).

### 2.1 Study participants

A total of 30 healthy male and female adults were intended to be enrolled in the study. Male and female subjects were eligible for inclusion if they were 18–65 years of age; had a body mass index (BMI) of 18 to <35.0 kg/m^2^; and weighed at least 110 lbs. Subjects were not eligible for inclusion if they had previous disorders of the GI tract; gastroenteritis in the 2 weeks prior to the study; diabetes; a history of drug or alcohol abuse; a previous diagnosis of neurological disorders, depression, or mental illness with psychosis; unexplained alarm features (e.g., fevers, blood in stools, unintentional weight loss greater than 10% of body weight in the last 3 months); used an antibiotic or any medication impacting gut transit during the 2 weeks prior to study; constipation or diarrhea; an allergy to tree nuts; a current pregnancy or were currently breastfeeding; or medical conditions affecting the pancreas, liver, thyroid, or gallbladder. Participants were enrolled in the study in groups of ten at a time.

### 2.2 Investigational product

Avela™, manufactured by Genomatica Inc., (San Diego, CA) is ≥ 99.7% chirally pure (*R*)-1,3-butanediol purified from microbial fermentation and is liquid at room temperature. To formulate one beverage serving, 11.5 g of Avela™ were mixed with distilled water to a total volume of 118 mL. Avela™ (*R*)-1,3-butanediol was consumed in the form of 3 servings of the beverage, with consumption of each serving separated by 30 min and each serving providing 11.5 g of (*R*)-1,3-butanediol [for a total intake of 34.5 g (*R*)-1,3-butanediol during the test day]. *(R)*-1,3-butanediol has Generally Recognized as Safe (GRAS) status for intakes of up to 34.5 g/day. This dosing regimen for Avela™ is based on 3 servings per day (up to 11.5 g per serving), which is similar to the proposed uses of another ketogenic substance, D-beta-hydroxybutyrate ester, the parent substance for *(R)*-1,3-butanediol. D-beta-hydroxybutyrate ester has received a no questions letter from the Food and Drug Administration on its GRAS status for use in bars, gels, and beverages at use levels up to 75 g/day over 2 to 3 servings per day.

### 2.3 Study design

This was an open-label, uncontrolled study, completed over a 5-h period on a single day. The study was completely virtual (i.e., participants were screened for eligibility, provided informed consent, were enrolled and completed the study in their own homes). Invitations to participate in the study were extended to employees of Genomatica Inc.; accordingly, several steps were taken to safeguard the rights and confidentiality of the participants, in line with recommendations provided by Resnick (2016). To ensure employees did not feel coerced to participate, a third-party clinical study coordinator was hired to oversee subject recruitment and the conduct of the study; as well, advertising for the clinical trial was “broad-based” and included mass company emails (as opposed to emails sent to individuals) and flyers posted in common areas of the company (e.g., the company kitchen/cafeteria). Those who were interested in participating in the study contacted the clinical study coordinator, who explained the requirements of the study and, for those who continued to be interested, scheduled a virtual meeting for obtaining informed consent and assessing subject eligibility.

All subjects included in the study were provided with study-related materials, including lancets, sharps container, alcohol swabs, Keto Mojo BHB monitor and ketone test strips (Keto-Mojo, Amsterdam, Netherlands), bandages, pregnancy test [if applicable], measuring tape, three 4-oz. (118-mL) bottles of sealed water premixed with Avela™ [each providing 11.5 g of (*R*)-1,3-butanediol], an ALOHA Organic Plant Based Protein Bar (Caramel Sea Salt), and study instructions.

All subjects included in the study were assigned to receive the active investigational product, Avela™ (*R*)-1,3-butanediol (i.e., as the study had a before-after study design, there was only one group in the study). Upon enrollment, the clinical research coordinator provided each participant with a unique identification number and a unique online portal link for study step-by-step instructions. Clinical trial data were recorded electronically, using a de-identified survey provided by Forsta.com, designed to protect subject anonymity (i.e., the survey was not affiliated with the subject’s name or any other personal information; rather, each participant used their unique identification code to access the survey and upload the results of the study, in real time).

Following a 12-h (overnight) fast and prior to ingesting any investigational product, participants completed, using the online portal, baseline assessments of GI symptomology (using a modified visual analogue scale [mVAS] GI Symptoms Tool) followed by sleepiness (using the Stanford Sleepiness Scale [SSS]). Each participant then measured their own baseline blood BHB level using a portable blood BHB monitoring device, the Keto-Mojo monitor (Keto-Mojo, Amsterdam, Netherlands), and recorded it using the online portal. Participants were then instructed to consume the first of three beverages prepared using Avela™ [each beverage contained 11.5 g of (*R*)-1,3-butanediol in 4 oz. (118 mL) water]. At the 30-min and 60-min time points, participants repeated the procedures followed at the baseline (0 min) time point; that is, they first completed the mVAS GI Symptoms Tool, followed by the SSS, and next, they measured and recorded their blood BHB levels, after which they consumed a second (at 30 min) and third (at 60 min) beverage prepared using Avela™. By 60 min, a total of 34.5 g of (*R*)-1,3-butanediol had been consumed. At each of the subsequent time points (90, 120, 180, 240, and 300 min), the mVAS GI Symptoms Tool and the SSS were completed, and blood BHB levels were measured and recorded. Of note, at 240 min, following the completion of the questionnaires and the blood BHB assessment, participants consumed one, 56-g ALOHA Organic Plant Based Protein Bar [Caramel Sea Salt, providing 236 kcal, 14 g of protein, 9 g of fat, and 25 g of carbohydrate (including 10 g of dietary fiber and 3 g of total sugars)], to provide sustenance to the study participants who, by 240 min, would have consumed nothing for 16 h (i.e., 12 h overnight and 4 h during the study), other than the investigational product.

**FIGURE 1:**
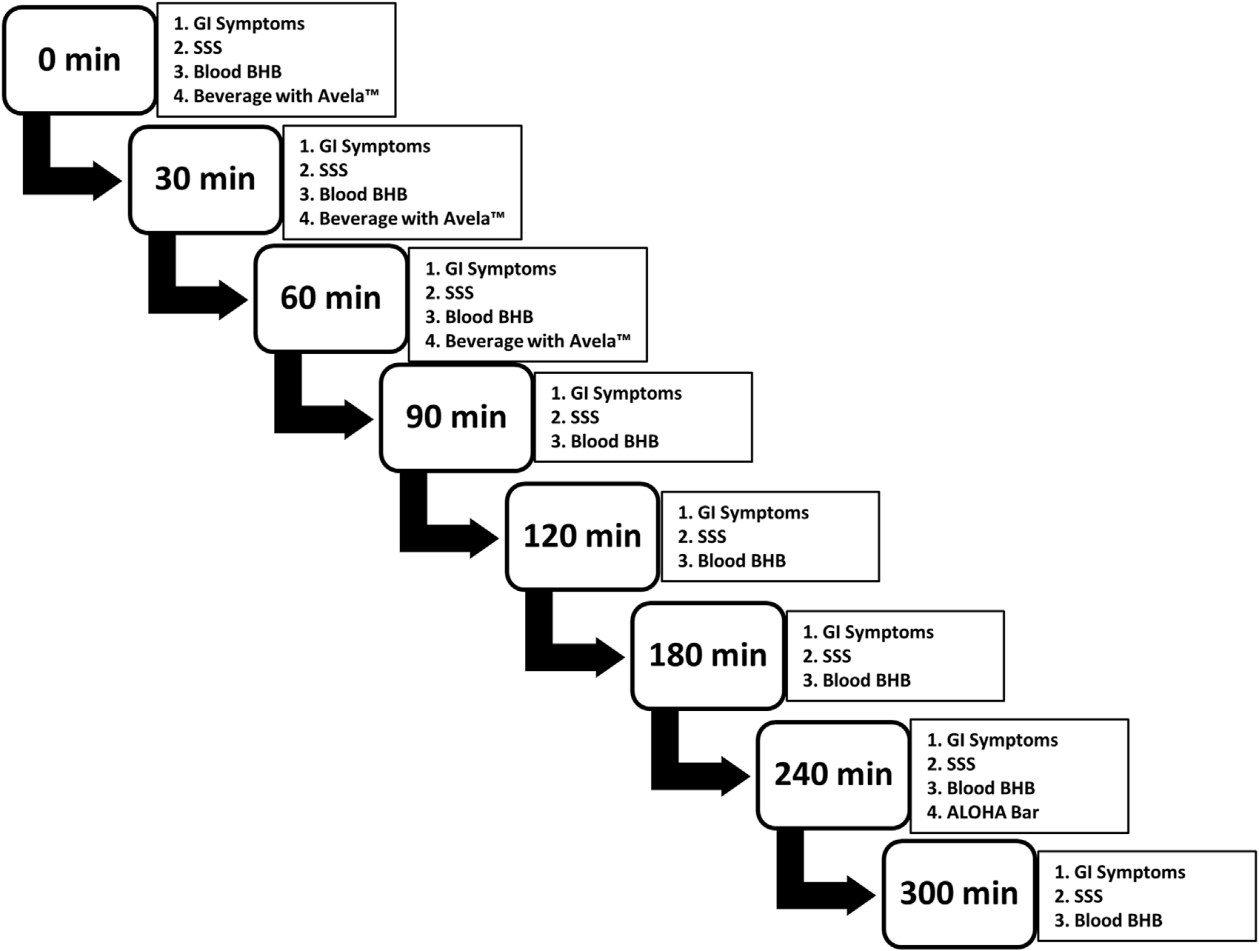
Study design and schedule of evaluations at each time point. Subjects performed each evaluation, in the order presented. Participants were provided with three beverages, each containing 11.5 g of Avela™ [(*R*)-1,3-butanediol] mixed with distilled water to a total volume of 118 mL; one beverage was consumed at 0 min, another at 30 min, and the final at 60 min, each after the completion of the questionnaires and the assessment of blood BHB levels. At 240 min, an ALOHA Organic Plant Based Protein Bar (Caramel Sea Salt) was consumed, again after the completion of the questionnaires and the assessment of blood BHB levels. GI symptoms were assessed using the modified visual analogue scale GI Symptoms Tool; sleepiness/alertness was assessed using the SSS; and blood BHB levels were assessed using the Keto Mojo BHB monitor and ketone test strips (Keto-Mojo, Amsterdam, Netherlands). Subjects completed the study at home and results were captured electronically, in real-time.BHB: beta-hydroxybutyrate; C_max_: maximum concentration; iAUC: incremental area under the curve; min: minute(s); SD: standard deviation; SSS, Stanford Sleepiness Scale.

Notably, during the electronic inputting of the study data, the participants were prompted to complete each task in the correct order so that the mVAS GI Symptoms Tool was always completed first, followed by the completion of the SSS, followed by the assessment of blood BHB levels. The correct schedule and order of events was maintained by controlling screen advances and the appearance of simple, one-step instructions during electronic data capture. The study design and schedule of evaluations at each time point are summarized in Figure 1.

### 2.4 Primary outcome: Blood BHB levels

The primary outcome of the study was the statistical difference in the blood BHB level at each time point relative to baseline, defined as time 0 min. To enable the assessment of blood BHB levels from fingertip capillary blood, each participant was provided with a Keto-Mojo monitor (Keto-Mojo, Amsterdam, Netherlands). Moore et al. (2021) tested the Keto-Mojo monitor for accuracy versus the Abbott Lab Precision Xtra glucose/ketone device (Abbott’s ketone meter) and reported there was excellent agreement between the two. Abbott’s ketone meter has been validated in comparison to mass spectrometry (the “gold standard” of ketone measurement) in animals (Pineda and Cardoso, 2015; Bach et al., 2016) and humans (Janssen et al., 2010; Yu et al., 2011) at ketone levels below 3 mM. The Keto-Mojo monitor is capable of measuring blood BHB levels from 0.1 to 8.0 mM; however, validity against the Abbott ketone meter at levels higher than 3.0 mM was not tested.

### 2.5 Secondary outcomes

The secondary outcomes of the study were the statistical differences in GI symptoms and sleepiness ratings at each time point relative to baseline, defined as time 0 min. GI symptoms were assessed using the mVAS GI Symptoms Tool and sleepiness was assessed using the SSS. Each of these tools is described further below.

#### 2.5.1 GI symptoms

The mVAS GI Symptoms Tool is a self-administered questionnaire that is modelled after the validated “gold standard” measurement tool predominantly used to test clinical gastroenterology scenarios (Bengtsson et al., 2013). Gaskell et al. (2019) tested the reliability of the mVAS in assessing GI symptoms during exercise, with and without dietary interventions. The mVAS GI Symptoms Tool includes a total of 19 symptoms, divided across four symptom groups, with the severity of each symptom scored out of 10, where 0 indicates absence of the symptom; 1 to 4 indicates the GI symptom is mild (i.e., sensation of GI symptom, but not substantial enough to interfere with activities); 5 to 9 indicates the GI symptom is moderate (i.e., GI symptom substantial enough to interfere with activities); and 10 indicates the GI symptom is severe (i.e., warranting cessation of all activities). The four symptom groups included upper GI symptoms, scored out of 70 (i.e., belching, heartburn, bloating, stomach pain, urge to regurgitate, regurgitation, projectile vomiting); lower GI symptoms, scored out of 50 (i.e., flatulence, lower abdominal bloating, urge to defecate, left intestinal pain, right intestinal pain); defecation, scored out of 40 (i.e., normal consistency, abnormal loose stools, diarrhea, bloody stools); and other GI symptoms, scored out of 30 (i.e., nausea, vomiting, stitch). A sample of the mVAS GI Symptoms Tool, which was completed at each time point of the study, is provided in Supplementary Table 1.

#### 2.5.2 Sleepiness

The SSS (Shahid et al., 2012) is a subjective tool to assess how sleepy a subject is feeling at specific moments in time. The scale requires respondents to select a rating of 1–7, where a “1” indicates the subject is “feeling active, vital, alert, or wide awake” and the highest score of “7” indicates the subject is “no longer fighting sleep, sleep onset soon; having dream-like thoughts” (Hoddes et al., 1973). The scale is validated, correlating with performance on the Wilkinson tests (r = 0.68). This SSS was completed at each time point of the study.

### 2.6 AEs

At each time point in the study, study participants were queried about AEs and were asked to describe and rate the severity of each using a five-point analog scale as mild (score of 1 or 2), moderate (score of 3 or 4), or severe (score of 5). Each AE was rated by the Principal Investigator as unrelated or possibly, probably, or definitely related to the investigational product.

### 2.7 Summary statistics and statistical analyses

#### 2.7.1 Summary statistics

All subjects who participated in the study were included in the analysis. Summary statistics (the number of observations, minimum, maximum, median, mean and standard deviation [SD]) were calculated for subject demographics, including BMI, height, weight, and age. Of note, BMI and age also were examined as categorical variables; specifically, the median age was used to classify participants as “younger” and “older,” and BMI was categorized as normal weight (18.5 to <25 kg/m^2^), overweight (25 to <30 kg/m^2^), or obese (≥30 kg/m^2^). Sex, age category (i.e., younger versus older), and BMI category (i.e., normal weight, overweight/obese) were presented as percentages.

The BHB mean, SD, median, minimum, and maximum at each time point are provided; in addition, the mean, SD, median, minimum, and maximum for the maximal BHB level (C_max_), the time at which the maximal BHB level was reached (T_max_), and the BHB iAUC over the 300-min study are provided.

For each of the four categories of GI symptoms, the mean, minimum, and maximum for each score are provided for each time point. Also, the proportion of subjects experiencing a maximum individual GI symptom score of 0, 1 to 4, 5 to 9, and 10, for each symptom over the 300-min study, after adjusting for (i.e., subtracting) the baseline pre-intake GI symptom severity rating from the post-intake GI symptom severity rating, is presented. As well, the number of unique GI symptoms experienced by each individual, after adjusting for baseline GI symptom severity ratings, is presented, as is the average percentage of time points at which each GI symptom was reported among those reporting the symptom.

For the SSS scores, the minimum, maximum, median, mean, and SD at each time point are provided, both before and after adjusting for baseline scores.

#### 2.7.2 Statistical analyses

All statistical analyses (described below) were conducted using SAS software (version 9.4, SAS Institute Inc., Cary, NC), and significance was defined as *p* < 0.05.

Mixed models with time (continuous variable) and time^2^ (continuous variable) as fixed effects and participants as random effects, were intended to be used to determine relationships with BHB levels, each of the 4 GI symptom category ratings, and the SSS scores; however, for each model, the residuals were not normally distributed, even after log transformation. Mixed models were then used to evaluate the effects of time (categorical variable) on the mean differences in BHB levels, the GI symptom category ratings, and the SSS scores, using the respective baseline values as references; however, the residuals were not normally distributed. For each model, the data were log transformed and the mixed models were refitted; however, the residuals still were not normally distributed. Of note, for the GI symptom category scores, there was an abundance of zeros (i.e., the median score for each of the four categories of GI symptoms was zero), and so the mixed model was determined to be inappropriate for the data. For comparisons of each post-baseline BHB level, GI symptom category score, and SSS score with the respective baseline (pre-intake) values, paired t-tests or Wilcoxon signed rank tests were conducted, as appropriate (i.e., if the data for the pair of values being compared were normal, then a paired t-test was used; otherwise, a Wilcoxon signed rank test was used). For the primary outcome only (i.e., differences in BHB levels at all time points versus 0 min), sensitivity analyses were conducted if there were any outliers (defined as any individuals with observations that were below the first quartile or above the third quartile by ≥ 1.5X the interquartile range [IQR]).

A correction for multiple comparisons (e.g., a Bonferroni correction) is normally applied to the secondary outcomes. However, as this was a pilot study, no correction for multiple comparisons was applied. Furthermore, the application of a Bonferroni correction to the statistical analysis of the secondary outcomes would have increased the difficulty in identifying possible GI intolerance or changes in sleepiness.

#### 2.7.3 Exploratory analyses

The effects of sex, age (categorical), and BMI (categorical) on BHB fasting level, iAUC, C_max_, and T_max_ were assessed using unpaired t-tests (with log transformation, if necessary) or the Wilcoxon Mann-Whitney test (if normality could not be achieved via log transformation). Correlations between age (continuous variable) and each of BHB fasting level, iAUC, C_max_, and T_max_, as well as BMI (continuous) and each of BHB fasting level, iAUC, C_max_, and Tmax, were assessed using Pearson’s correlation coefficient (or Spearman’s correlation coefficient, if a non-parametric test was more appropriate). As well, at each time point, correlations between blood BHB levels and scores for each GI symptom group as well as between blood BHB levels and SSS scores, were assessed using Pearson’s correlation coefficient (or Spearman’s correlation coefficient, if a non-parametric test was more appropriate).

## 3 Results

### 3.1 Participant demographics

The goal was for 30 participants to be enrolled in the study; however, only 26 participants were enrolled, given that recruitment was slower than had been anticipated. Participant demographics are summarized in Table 1. Of the 26 participants, 53.9% were males and 46.1% were females. The study participants had a mean age of 42.5 years, and a mean BMI of 25.53 kg/m^2^. Of the 26 participants, 12 (46.2%) were normal weight (BMI was 18.5 to <25 kg/m^2^), 12 (46.2%) were overweight (BMI was 25 to <30 kg/m^2^), and two (7.6%) were obese (BMI ≥30 kg/m^2^). Since there were only two obese participants, results from these individuals were included with those categorized as overweight in all analyses in which BMI was assessed as a categorical variable, resulting in two categories (i.e., “normal weight” versus “overweight or obese”). The median age of 42.50 years was used to separate study participants as “relatively younger” (<42.50 years) and “relatively older” (≥42.50 years).

**TABLE 1:** Participant demographics.

	n	Median	Mean	SD	Min	Max
BMI (kg/m^2^)	26	25.29	25.53	3.53	19.01	33.75
Height (m)	26	1.73	1.73	0.10	1.57	1.96
Weight (kg)	26	71.44	76.50	15.38	55.34	106.59
Age (y)	26	42.50	42.50	11.97	25.00	61.00
Male sex, n (%)	14 (53.9%)	--	--	--	--	--
Female sex, n (%)	12 (46.1%)	--	--	--	--	--

BMI: body mass index; Max: maximum; Min: minimum; n: number of individuals; SD: standard deviation; y: years.

### 3.2 Blood BHB results

Blood BHB levels following the 12-h overnight fast (i.e., at 0 min) and at the subsequent time points are presented in Table 2. At baseline, the mean blood BHB level was 0.23 ± 0.21 mmol/L. It is notable that one study participant had a fasting blood BHB level of 1.10 mmol/L; after following-up with this subject, it was indicated that they habitually follow a low-carbohydrate diet, though not a strict ketogenic diet. The mean C_max_, which was 2.10 ± 0.97 mmol/L, was reached at an average of 133.85 ± 57.07 min, approximately 1 h following the consumption of the final beverage containing Avela™ (*R*)-1,3-butanediol. One individual reached a C_max_ of 5.5 mmol/L at 180 min; this was not the same individual who had a high fasting BHB level of 1.10 mmol/L at baseline. The next two highest C_max_ levels of 3.9 and 3.1 mmol/L were reached at 240 and 180 min, respectively. The average blood BHB iAUC_0–300 min_ was 376.73 ± 156.76 mmol/L*min. At 300 min (i.e., 1 hour following the consumption of the ALOHA Plant Based Protein Bar), the average blood BHB level was 1.11 ± 0.85 mmol/L.

**TABLE 2:** Blood BHB levels over the 300-min study. ^a^, ^b^.

Time (minutes)	Mean	SD	Median	Min	Max
0	0.23	0.21	0.20	0.10	1.10
30	1.00	0.29	1.00	0.50	1.70
60	1.47	0.49	1.40	0.60	2.90
90	1.81	0.54	1.75	1.00	3.10
120	1.83	0.62	1.70	0.70	3.60
180	1.82	0.99	1.50	0.70	5.50
240	1.50	0.83	1.10	0.60	3.90
300	1.11	0.85	0.80	0.30	3.70
iAUC	376.73	156.76	336.00	154.50	828.00
C_max_	2.10	0.97	1.90	1.00	5.50
T_max_	133.85	57.07	120.00	60.00	240.00

BHB: beta-hydroxybutyrate; C_max_: maximum concentration; iAUC: incremental area under the curve; Max: maximum; Min: minimum; n: number of individuals; SD: standard deviation; T_max_: time at which the C_max_ was observed.

^a^ The units are mmol/L, except for iAUC, for which the unit is mmol/L*min.

^b^ For two participants, the baseline BHB, levels were clearly entered in error (i.e., 103 mmol/L and 72 mmol/L). Thus, these baseline BHB, levels were replaced with the average baseline BHB, level, computed using data from the remaining 24 participants (i.e., 0.2 mmol/L). In addition, for one of these participants, the BHB, level entered at 30 min was 4.0 mmol/L. This was assumed to be an error, and the value was replaced with the average BHB, level at 30 min from the remaining 25 participants, which was 1.0 mmol/L.

**FIGURE 2:**
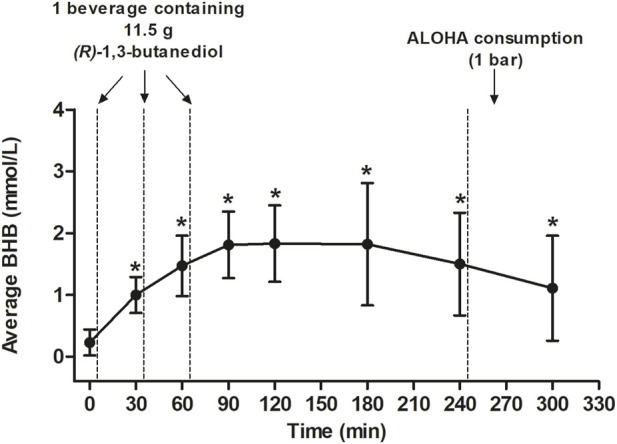
Average BHB levels (mmol/L) following consumption of three beverages made with Avela™ [each containing 11.5 g (R)-1,3-butanediol] at approximately 0, 30, and 60 min after the assessment of baseline BHB levels. An ALOHA Organic Plant Based Protein Bar (Caramel Sea Salt) was consumed after the completion of questionnaires and the measurement of BHB at the 240-min time point. All values are mean ± SD. An asterisk indicates a significant difference (*p* < 0.05) at that time point, relative to baseline (0 min). The *p*-values at 30, 90, and 120 min were calculated using paired t-tests. The *p*-values at 180, 240, and 300 min were calculated using the Wilcoxon signed rank test. The mean iAUC was 376.73 ± 156.76 mmol/L x min and the mean Cmax was 2.10 ± 0.97 mmol/L which was reached after an average of 133.85 ± 57.07 min. BHB, betahydroxybutyrate; C_max_, maximal concentration; GI, gastrointestinal; iAUC, incremental area under the curve; min, minute(s); SD, standard deviation.

Each post-baseline blood BHB measurement (i.e., at 30, 60, 90, 120, 180, 240, and 300 min) was significantly greater than that at baseline (Figure 2). Seven individuals were identified as outliers, based on the definition of an outlier as an individual with an observation that was below the first quartile or above the third quartile by ≥ 1.5X the IQR. Despite the removal of these seven individuals from the analysis, BHB levels assessed at 30, 60, 90, 120, 180, 240, and 300 min were still each significantly greater than at 0 min (data not shown).

Although the design of the study does not allow for an assessment of whether or not the consumption of the plant-based protein bar at 240 min had any impact on ketone levels thereafter, in an unplanned analysis, the slopes of the lines between 180 and 240 min (the hour before the bar was consumed) and 240 and 300 min (the hour after the bar was consumed) were compared using a Student’s paired *t*-test. Although the slope of the line between 240 and 300 min (−0.39 ± 0.31 mmol/L over 60 min) was slightly steeper than that between 180 and 240 min (−0.32 ± 0.60 mmol/L over 60 min), there was no significant difference in the slopes of the lines (*p* = 0.50).

### 3.3 GI symptomology results

The maximum severity reported by each individual for each GI symptom over the 300-min study, after adjusting for the baseline symptom severity rating, is shown in Table 3. For one participant, the baseline-adjusted maximum scores for urge to regurgitate, nausea, and dizziness were 9, 9, and 8, respectively. This participant tested positive for COVID-19 the next day. For all other participants, the maximum severity score for each GI symptom was either 0 (indicating no symptoms) or 1 to 4 (mild). The most commonly reported GI symptoms were mild belching, mild nausea, and mild dizziness, each of which was reported by 8, 4, and 9 participants, respectively.

**TABLE 3:** Baseline-adjusted maximum GI symptom severity rating.

	≤0 (None)	1 to 4 (Mild)	5 to 9^a^ (Moderate)	10 (Severe)
Belching	18	8	--	--
Heartburn	25	1	--	--
Bloating	25	1	--	--
Stomach pain	24	2	--	--
Urge to regurgitate	24	1	1	--
Regurgitation	26	--	--	--
Projectile vomiting	26	--	--	--
Flatulence	25	1	--	--
Lower abdominal bloating	26	--	--	--
Urge to defecate	25	1	--	--
Left intestinal pain	26	--	--	--
Right intestinal pain	25	1	--	--
Normal consistency	26	--	--	--
Abnormal loose stool consistency	26	--	--	--
Diarrhea	26	--	--	--
Bloody stools	26	--	--	--
Nausea	21	4	1	--
Dizziness	16	9	1	--
Stitch	26	--	--	--

GI: gastrointestinal.

^a^ All the symptoms rated as moderate (i.e., urge to regurgitate, nausea, and dizziness) were reported by the same participant who tested positive for COVID-19, the next day.

**FIGURE 3:**
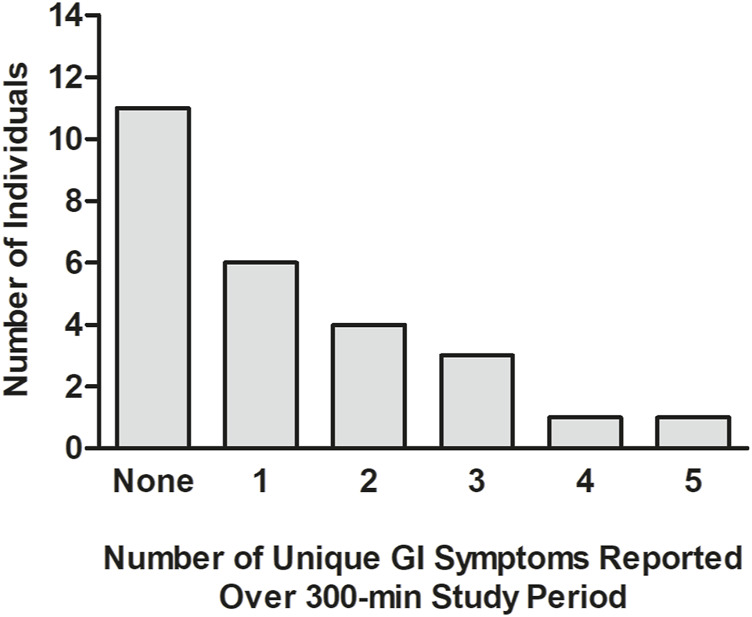
Number of different GI symptoms reported by each individual during the 300-min study. The analysis was undertaken after adjusting each post-baseline GI symptom rating for that reported at baseline (time = 0 min), prior to the consumption of (*R*)-1,3-butanediol.GI: gastrointestinal; min: minute(s).

In Figure 3, the number of different GI symptoms reported over the 300-min study by each individual, after adjusting for baseline symptom severity, is shown. Of the 26 participants, 11 (42.3%) did not report any GI symptoms, six (23.1%) reported one mild GI symptom, four (15.4%) reported two mild GI symptoms, three (11.5%) reported three mild GI symptoms, one (3.8%) reported four mild GI symptoms, and one (3.8%) (the same individual who tested positive for COVID-19 the next day) reported five GI symptoms, three of which were moderate and two of which were mild. In Table 4, the average percentage of time points at which each GI symptom was reported among individuals reporting the symptom is presented. After adjusting for baseline GI symptom scores, one individual reported mild heartburn at all 7 post-baseline time points, and each of the other GI symptoms was reported at an average of 14.3%–50.0% of the time points.

**TABLE 4:** Average percentage of time points at which each GI symptom was reported among individuals reporting the symptom.^a^

GI Symptom	Average Percentage of Time Points^b^
Belching	35.7% (n = 8)
Heartburn	100% (n = 1)
Bloating	42.9% (n = 1)
Stomach pain	21.4% (n = 2)
Urge to regurgitate	50.0% (n = 2)
Regurgitation	--
Projectile vomiting	--
Flatulence	14.3% (n = 1)
Lower abdominal bloating	--
Urge to defecate	14.3% (n = 1)
Left intestinal pain	--
Right intestinal pain	14.3% (n = 1)
Normal consistency	--
Abnormal loose stool consistency	--
Diarrhea	--
Bloody stools	--
Nausea	28.6% (n = 5)
Dizziness	41.4% (n = 10)
Stitch	--

GI: gastrointestinal.

^a^ Analysis was conducted on baseline-adjusted GI, symptoms, and so the 0-min time point was not included.

^b^ As an example of how this table is intended to be interpreted, heartburn was experienced by 1 person at 7/7 (100%) of the post-baseline time points, and belching was experienced by 8 participants at an average of 2.5/7 (35.7%) of the post-baseline time points.

**FIGURE 4:**
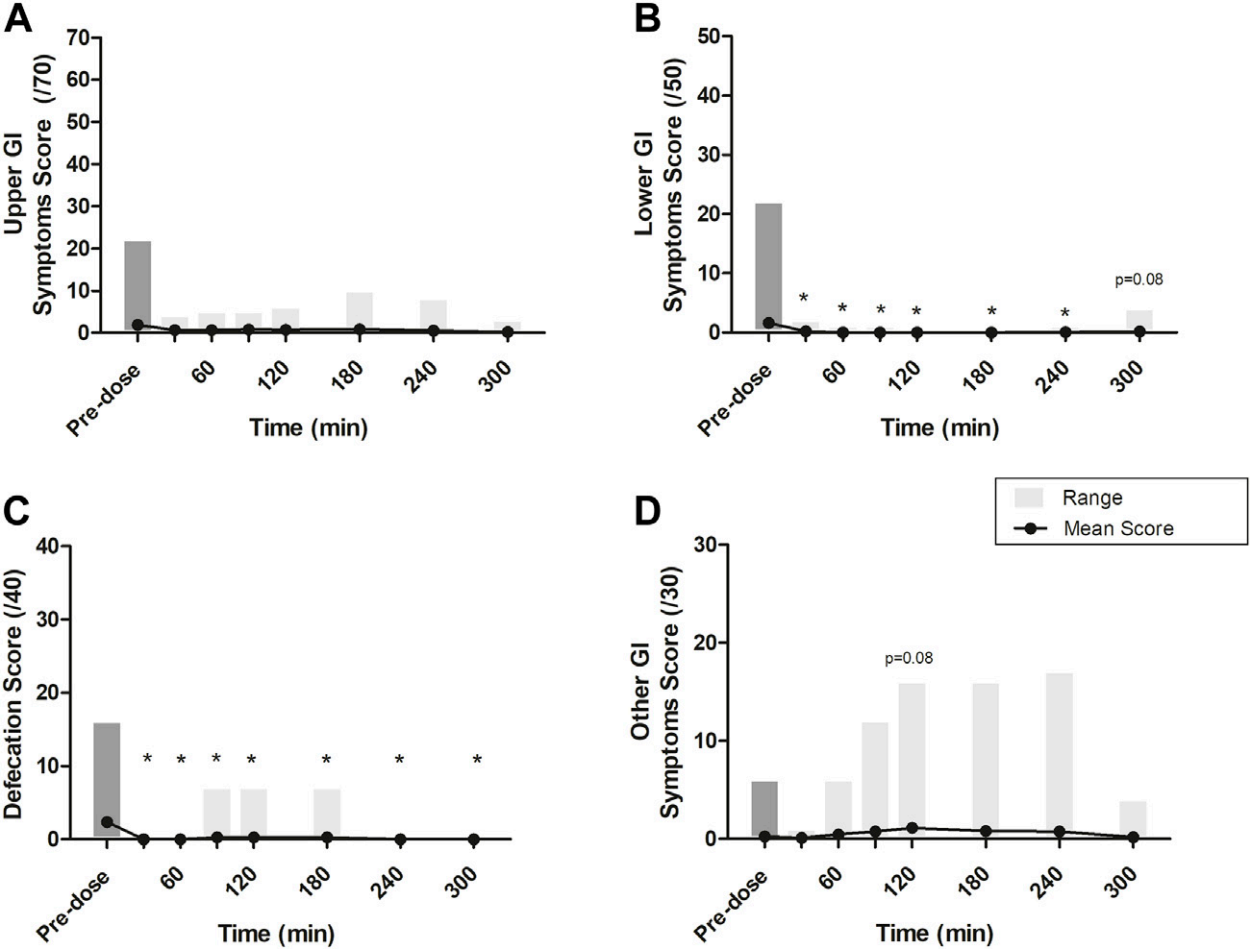
Mean scores at each time point across four GI symptom groups: **(A)** Upper GI symptoms, scored out of 70 (i.e., belching, heartburn, bloating, stomach pain, urge to regurgitate, regurgitation, projectile vomiting); **(B)** Lower GI symptoms, scored out of 50 (i.e., flatulence, lower abdominal bloating, urge to defecate, left intestinal pain, right intestinal pain); **(C)** Defecation, scored out of 40 (i.e., normal consistency, abnormal loose stools, diarrhea, bloody stools); and **(D)** other GI symptoms, scored out of 30 (i.e., nausea, dizziness, stitch). Grey boxes represent the range of scores reported at each time point. All *p*-values were determined using the Wilcoxon Mann-Whitney test. * represents statistically significant relative to time 0 min (*p* < 0.05). At 0, 30, and 60 min, after the completion of questionnaires and the assessment of BHB levels, a beverage made with Avela™ [containing 11.5 g (R)-1,3-butanediol] was consumed. At 240 min, after the completion of questionnaires and the assessment of BHB levels, an ALOHA Organic Plant Based Protein Bar (Caramel Sea Salt) was consumed. BHB: beta-hydroxybutyrate; BMI: body mass index; C_max_: maximum concentration; iAUC: incremental area under the curve; min: minute(s); NW: normal weight; OW: overweight; T_max_: time at which the C_max_ is observed.

Global scores (including the means and ranges) for upper GI symptoms, lower GI symptoms, defecation, and other GI symptoms at each time point are presented in Figure 4. The global score medians were zero at all time points, and, for upper GI symptoms, lower GI symptoms, and defecation, the greatest mean scores were recorded at baseline. One subject who had a normal bowel movement at baseline (pre-dosing) mistakenly reported bowel movements at 3 additional time points. Only for the “Other GI symptoms” score was the mean score at baseline not greater than post-dosing. At 120 min, the score for the other GI symptoms category (which includes nausea, dizziness, and stitch) was nearly significantly greater than at baseline (*p* = 0.08). No participants reported stitch; thus, the increase in post-baseline measures was due to the reporting of nausea and dizziness, each by 5 and 10 participants, respectively.

The ratings by individual subjects for these symptoms over the course of the 300-min study, after adjusting for baseline ratings, is shown in Supplementary Table S2 **.**

### 3.4 Sleepiness results

Unadjusted and baseline-adjusted SSS scores at each time point are summarized in Table 5. None of the SSS scores post-baseline were significantly different from that assessed at baseline.

**TABLE 5:** Unadjusted and baseline-adjusted SSS scores.

Time (minutes)	Absolute SSS Scores					Baseline-adjusted SSS Scores				
	Median	Mean	SD	Min	Max	Median	Mean	SD	Min	Max
0	2.5	2.08	0.98	1	3	--	---	---	---	---
30	2.0	1.92	0.93	1	4	0	−0.15	0.61	−2	1
60	1.0	1.69	0.84	1	3	0	−0.38	1.02	−2	2
90	2.0	1.92	1.06	1	4	0	−0.15	1.32	−2	3
120	2.0	1.96	1.00	1	4	0	−0.12	1.31	−2	2
180	2.0	2.00	0.94	1	4	0	−0.08	1.16	−2	2
240	2.0	2.00	1.23	1	5	0	−0.08	1.26	−2	2
300	1.0	1.62	0.85	1	3	0	−0.46	1.17	−2	2

Max: maximum; Min: minimum; SD: standard deviation; SSS: Stanford Sleepiness Scale.

### 3.5 Results of exploratory analyses

**FIGURE 5:**
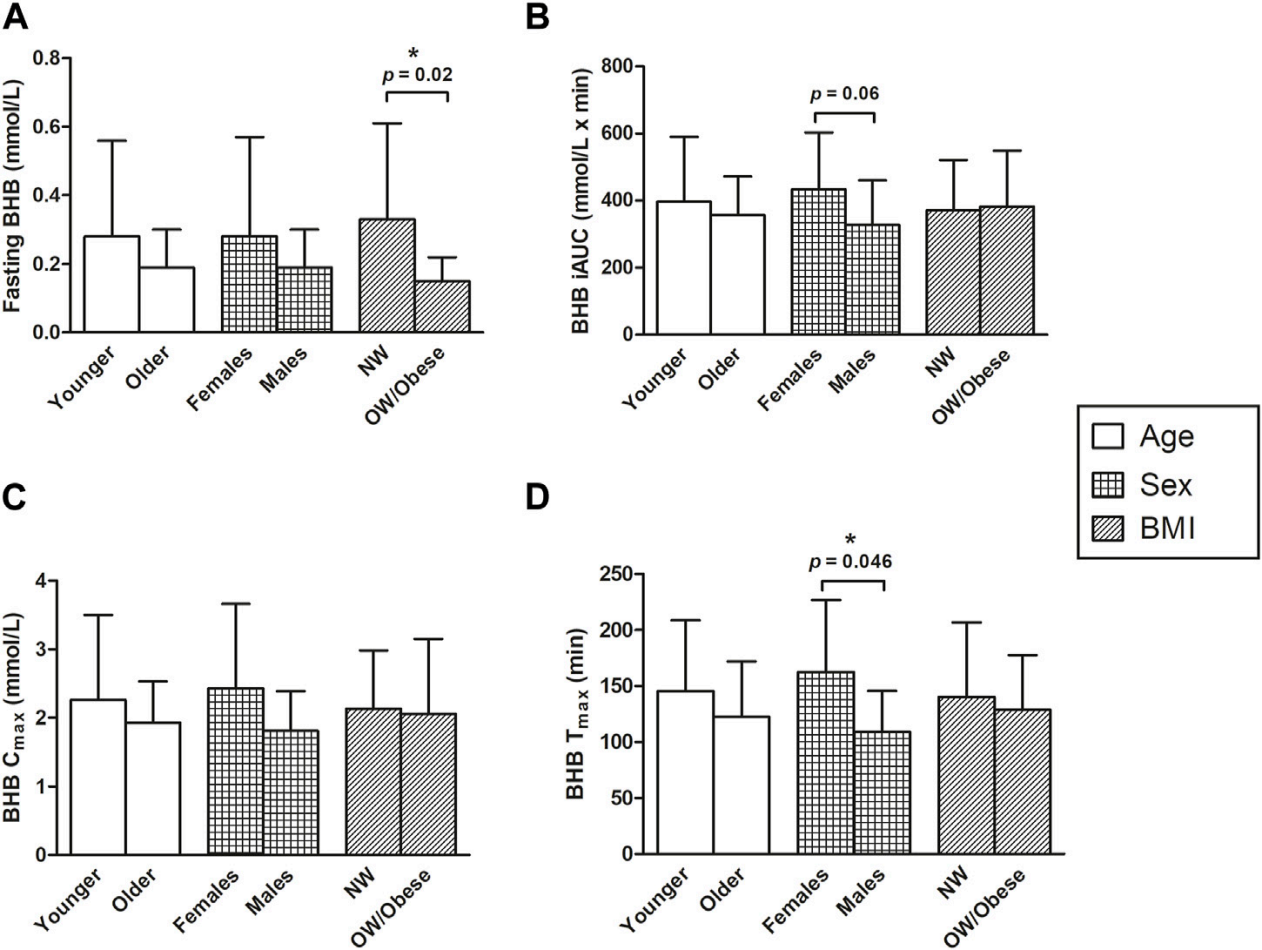
The effects of sex, age, and BMI on **(A)** fasting BHB levels (mmol/L) **(B)** BHB iAUC (mmol/L x min) **(C)** Cmax levels (mmol/L), and **(D)** Tmax levels (min). An asterisk indicates a significant difference (*p* < 0.05) between groups (i.e., younger vs. older, females vs. males, NW vs. OW/obese). **(A)** All *p*-values were determined using the Wilcoxon Mann-Whitney test. Normal weight subjects had greater fasting BHB levels, compared to overweight and obese subjects (*p* = 0.02). **(B)** Two-sample t-tests were applied for comparing the mean differences between BHB iAUC in the two age groups and log transformation for comparing mean differences between BHB iAUC in males vs. females; and the *p*-value for BMI was calculated using the Wilcoxon Mann-Whitney test. There was a trend towards greater BHB iAUCs in females, compared to males; however, this association was not significant (*p* = 0.06). **(C)** The BHB Cmax values were log-transformed for comparing the sex and age effect and then the two-sample t-tests were calculated. The *p*-value for BMI was calculated using the Wilcoxon Mann-Whitney test. **(D)** The Tmax values for the effects of age and BMI were log-transformed and then compared using the two-sample t-test. The Wilcoxon Mann-Whitney test was applied to compare the difference between the mean Tmax of males and females. The time for females to reach their maximum concentration of BHB was significantly longer than that of males (162.50 ± 64.54 vs. 109.29 ± 36.47 for females and males, respectively; *p* = 0.046). BHB, beta-hydroxybutyrate; BMI, body mass index; C_max_, maximum concentration; iAUC, incremental area under the curve; NW, normal weight; OW, overweight; T_max_, time at which the C_max_ is observed.

**FIGURE 6:**
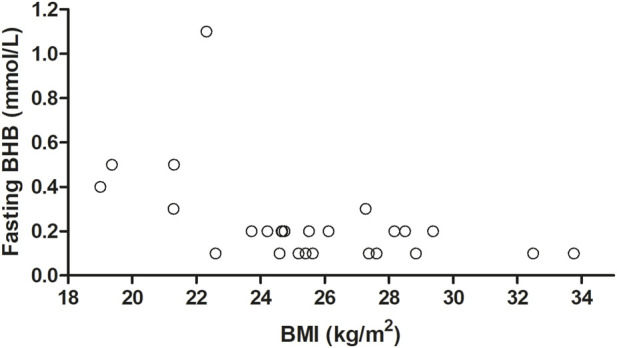
The effect of BMI as a continuous variable on fasting BHB levels. Fasting BHB levels were negatively associated with BMI (r^2^ = −0.56; *p* = 0.003), as calculated using Spearman’s correlation. BHB, betahydroxybutyrate; BMI, body mass index; r^2^ correlation coefficient.

The associations of age, BMI, and sex with fasting BHB levels, C_max_, T_max_, and iAUC_0–300 min_ are shown in Figure 5 (where age and BMI were treated as categorical variables) and Table 6 (where age and BMI were treated as continuous variables). Fasting BHB levels were significantly greater in individuals classified as normal weight relative to those classified as either overweight or obese (Figure 5A; *p* = 0.02); likewise, fasting BHB levels were significantly negatively associated with BMI, with a Spearman’s correlation coefficient of −0.56 (*p* = 0.003) (Figure 6). BHB T_max_ values were nearly significantly and inversely associated with BMI (Spearman’s correlation coefficient of −0.36; *p* = 0.07; Table 6). In females, BHB T_max_ was significantly greater than in males (*p* = 0.046) (Figure 2D). Also in females, BHB iAUC_0–300 min_ was nearly significantly greater than in males (*p* = 0.06). In unplanned analyses, the associations between body weight and each of fasting BHB, BHB iAUC_0–300 min_, BHB C_max_, and BHB T_max_ also were evaluated. Body weight was inversely and significantly or nearly significantly associated with each of these variables (Spearman’s correlation coefficients of −0.39, −0.45, −0.39, and −0.58, respectively, and associated *p*-values of 0.047, 0.020, 0.050, and 0.002, respectively) (Table 6). An unpaired *t*-test was used to evaluate the difference in body weight between sexes and, not surprisingly, body weight was significantly lower in females than in males (*p* = 0.0096). Interestingly, when we looked at the difference in BMI between males and females, there was no significant difference (*p* = 0.2184). Significant relationships between blood BHB levels and each of the GI symptom category scores, as well as between blood BHB levels and the SSS scores, were not identified at any of the time points examined. Adjusting for body weight had no impact on these results (data not shown).

**TABLE 6:** Relationships between age, BMI or weight and fasting BHB, BHB iAUC, BHB C_max_, and BHB T_max_.

	Age ^1^		BMI ^1^		Weight ^1^	
Outcome	**r** ^**2**^	***p*-value** ^**2**^	**r** ^**2**^	***p*-value** ^**2**^	**r** ^**2**^	***p*-value** ^**2**^
Fasting BHB	−0.16	0.42	−0.56	0.003	−0.39	**0.047**
BHB iAUC	0.12	0.57	−0.20	0.32	−0.45	**0.020**
BHB C_max_	0.09	0.68	−0.22	0.29	−0.39	0.050
BHB T_max_	0.04	0.65	−0.36	0.07	−0.58	**0.002**

BHB: beta-hydroxybutyrate; BMI: body mass index; C_max_: maximum concentration; iAUC: incremental area under the curve; r^2^: correlation coefficient; T_max_: time at which the C_max_ is observed.

^1^ Calculated using Spearman’s correlation.

^2^ Bolded *p*-values are statistically significant.

### 3.6 Unsolicited AEs

Spontaneous AEs were not reported at baseline, 30 or 60 min. The most frequently reported AE was headache, reported by five subjects and judged by the Principal Investigator to be possibly related to the study product for two persons. One of the individuals reported a persistent mild (score of 1) headache at 90, 120, 180, 240, and 300 min, with no other AEs reported. The second individual reported a mild headache (score of 1) at 180, 240 and 300 min. Of the three remaining persons, the headache was attributed to a positive COVID-19 diagnosis the following day (n = 1), fasting (n = 1), or resolved after one rating (n = 1). A feeling of warm and sweating was reported by one individual at 90 and 120 min; these symptoms, which disappeared within 60 min of a drink of water, were judged by the Principal Investigator to be possibly related to the study product.

## 4 Discussion

In the study reported herein, three servings of a beverage containing Avela™ and each providing 11.5 g of (*R*)-1,3-butanediol were consumed in close succession at 0, 30, and 60 min by participants in a fasted state, and effects on BHB levels, GI symptomology, and sleepiness were assessed over a total period of 300 min.

Consumption of three servings of (*R*)-1,3-butanediol (11.5 g, each; 34.5 g, total) resulted in a mean blood BHB C_max_ of 2.10 ± 0.97 (SD) mmol/L, which was achieved roughly 1 h post consumption of the final beverage containing Avela™. In a recent publication, 12 healthy individuals who consumed a single serving of 10 g of (*R*)-1,3-butanediol following an 8-h fast achieved a mean peak blood BHB level of 1.2 ± 0.3 mmol/L (SD) approximately 40 min post consumption (Falkenhain et al., 2023). In the current study, the mean blood BHB iAUC_0–300 min_ was 376.73 ± 156.76 (SD) mmol/L*min, a value approximately triple that which was measured (iAUC_0–240 min_ of 118 ± 64 mmol/L*min) following the consumption of 10 g (*R*)-1,3-butanediol in the study by Falkenhain et al. (2023). This is consistent with total intakes administered in each study. Blood BHB levels appear to continue to rise with each subsequent consumption of a beverage prepared with Avela™ (Figure 2) to a peak (roughly 1 h following the last consumption of Avela™), followed by a slow decline to the end of the study, where the mean level was 1.11 ± 0.85 mmol/L. Consumption of a plant-based protein bar (providing 25 g carbohydrate, 14 g protein, and 9 g fat) at 240 min did not appear to change the kinetics of BHB disappearance in the 1 h post-consumption. In a study by Cuenoud et al. (2020) plasma BHB levels were elevated following the consumption of 12 g of D-BHB (in the form of salts), despite the consumption, 30 min after the D-BHB salts beverage, of a breakfast meal (consisting of 2 boiled eggs, 2 pieces of toast, 1 slice of cheese, and 1 portion of fruit jam, providing a total of 423 kcal, 20 g fat, 24 g protein, and 32 g carbohydrate). Thus, based on preliminary evidence, BHB levels achieved via exogenous ketone sources are not affected by the consumption of foods, but additional studies are needed to better understand this. Overall, maintenance of elevated levels of BHB in the blood for at least 5 h using exogenous administration appears quite feasible.

Statistically significant inverse associations between BMI and fasting BHB and between body weight and each of fasting BHB, BHB iAUC_0–300 min_, BHB C_max,_ and BHB T_max_ are interesting. Body weight may be a more sensitive predictor of ketogenic potential than BMI. Increased adiposity is associated with increased insulin, which potently inhibits lipolysis and ketogenesis (Horowitz et al., 1999; Horowitz et al., 2001). Indeed, in our study, of the 6 subjects with a BMI ≤23 kg/m^2^, 5 had a fasting plasma BHB level greater than 0.2 mmol/L (of the remaining 19 subjects with a BMI >23 kg/m^2^, 18 had a fasting plasma BHB level less than 0.2 mmol/L).

Additionally, the significantly greater BHB T_max_ (*p* = 0.046) and nearly significantly greater BHB iAUC_0–300 min_ (*p* = 0.06) determined for females versus males also are interesting, given that sex differences have been reported in metabolic changes following fasting. Increases in plasma fatty acid and ketone body concentrations (Merimee and Fineberg, 1973; Merimee et al., 1978; Haymond et al., 1982) with a concomitant decrease in blood glucose concentration (Merimee and Fineberg, 1973; Haymond et al., 1982) which normally occur during short-term fasting (60–86 h), are reportedly greater in women than in men (Mittendorfer et al., 2001). While effects of gender may be confounded by effects of adiposity (given that men typically have greater body weights than females), in a fasting study in which males and females were matched for body fat percentage, the mean plasma fatty acid and glycerol concentrations were higher in women than in men (following a 14-h fast), and continued to increase with duration of fasting (22-h fast); however, glucose concentrations were similar between sexes (Mittendorfer et al., 2001). Although the mechanisms for these differences are not known, Mittendorfer et al. (2001) hypothesized that they may be related to differences in major plasma hormones that regulate lipolysis.

To further evaluate the effects of body weight on fasting BHB, BHB C_max_, BHB T_max_, and BHB iAUC, we examined associations within each gender, since body weight was significantly lower in females than in males. Although all Spearman correlation coefficients were negative, only those for fasting BHB and BHB T_max_ were significant, and only for females (data not shown). Of course, the sample sizes are very small (14 males and 12 females) and so these results should be interpreted with caution. Additional studies are needed to better understand the effects of gender and body weight on ketone levels.

GI symptomology was assessed at multiple time points throughout the study to capture the presence or absence, as well as the severity, of 19 symptoms. Overall, (*R*)-1,3-butanediol was well-tolerated with no or mild GI symptoms reported by most participants. Post-baseline adjusted symptoms were rated as moderate by only one person, for each of urge to regurgitate, nausea and dizziness; this person tested positive for COVID-19 the following morning. The GI symptoms that were most frequently reported were mild belching, mild nausea, and mild dizziness, each of which was reported by eight, four, and nine persons, respectively, along with mild headache reported by 5 subjects. Past studies have reported diarrhea, decreased appetite, and headache with KS (Leckey et al., 2017; Waldman et al., 2018; Stubbs et al., 2019) or KME (Clarke et al., 2012). Stubbs et al. (2019) suggested that the salt bolus consumed with KS may affect fluid homeostasis in the gut leading to symptoms not observed in our study with (*R*)-1,3-butanediol. While symptoms of nausea and dizziness/light headedness have been reported following consumption of other ketone beverages (Clarke et al., 2012; Leckey et al., 2017; Vandoorne et al., 2017; Evans and Egan, 2018; Evans et al., 2018; Waldman et al., 2018; Stubbs et al., 2019), factors that may have contributed to these effects could be prolonged fasting (Torelli and Manzoni, 2010) and/or caffeine withdrawal (Sajadi-Ernazarov et al., 2022). Indeed, in our study, the subjects were fasted overnight and completed the study in a fasted state (until the 240 min mark, when the ALOHA bar was consumed). The maximum ratings of dizziness were at 120 and 240 min. Once the ALOHA bar was consumed (240 min), the dizziness ratings returned to baseline values in most subjects, thereby suggesting that the dizziness experienced may have been attributed to prolonged fasting, rather than the consumption of (R)-1,3-butanediol. In addition, it is noteworthy that the participants had an intense schedule of evaluations during the 5-h study, which entailed the completion of questionnaires, finger-pricking (which is nauseating for some people), and entering data electronically, repeatedly. Finally, *(R)*-1,3-butanediol has a bitter taste which may have contributed to the nausea. Of note, neither “other GI Symptoms” (which included nausea and dizziness) nor any of the other GI symptom categories was associated with BHB levels, even after adjusting for body weight (data not shown).

No differences in the reporting of sleepiness post-baseline for any time points in the study were observed. Some scientists suggest that consumption of a ketogenic diet or exogenous ketones can improve sleep patterns, cognition and outcomes in neurologic conditions (reviewed in Hallböök et al., 2012; Murray et al., 2016; Evans and Egan, 2018). Additionally, others hypothesize a potential link between low-carbohydrate diets and feelings of euphoria (Brown, 2007). For this reason, an additional questionnaire was provided to subjects in the study herein to determine whether there was an effect on sleepiness following consumption of the study product. The SSS has the greatest reliability and validity in predicting performance on tasks related to alertness (e.g., reaction time, vigilance tests) following total sleep deprivation (Shahid et al., 2012). The SSS likely lacked the sensitivity to identify any changes in alertness in this study, but was selected given its simplicity of use (it is a 7-point Likert scale) and in consideration of the design of the study (at-home, conducted by the participants themselves). The study reported herein was an acute feeding study; longer-term feeding trials in patients with measurable disorders may be more appropriate for assessing the neurocognitive effects of ketones and effects on sleepiness (García-Rodríguez and Giménez-Cassina, 2021).

Several limitations were inherent to this study and warrant discussion. The study was conducted in the participants’ homes and the results of the study were contingent on the participants performing the trial, as intended. Subjects followed step-by-step prompts on a screen to mitigate issues that might arise with subject error in study conductance; they were prompted to advance to a subsequent step only after the prior step was completed. This helped to ensure that all study-related procedures were completed in the proper order at each time point. The Keto-Mojo monitor was utilized to measure blood BHB levels. This monitor is capable of measuring ketones from 0.1 mmol/L to 8.0 mmol/L; however, validity at detecting BHB levels greater than 3.0 mmol/L was not tested. In this study, there were 208 BHB measurements across all 26 participants and time points; of these, 8 were greater than 3 mmol/L. Of note, plasma butanediol levels were not measured and so, it is unknown at what level they circulate in the body; this could be measured in a future study. The requirement for a minimum 12-h fast and the administration of the study product in three servings in close succession (each separated only by a half-hour) on an empty stomach, without food, was considered to represent a best-case scenario in terms of increasing BHB levels, but a worst-case scenario regarding GI tolerability.

## 5 Conclusion

In this study, peak blood BHB levels of 2.10 ± 0.97 mmol/L were achieved roughly 1-h post consumption of the last (*R*)-1,3-butanediol beverage, when a total of 34.5 g (*R*)-1,3-butanediol would have been consumed. (*R*)-1,3-butanediol was well tolerated. Unlike reports following consumption of KME and KS, no subjects reported experiencing diarrhea following consumption of the study product. The most frequently reported symptoms of belching, nausea, headache and dizziness, were mild and transient in nature. No effect on subject sleepiness was reported.

This study demonstrates the feasibility of administering oral (*R*)-1,3-butanediol to achieve blood BHB levels typical of physiologic ketosis. There is a high likelihood of creating regimens for chronic administration which could potentially sustain a desired level throughout the day. Next steps require testing of such regimens in patients with measurable disease or performance endpoints.

## Data Availability

The raw data supporting the conclusion of this article will be made available by the authors, without undue reservation.
