# Supplementary material for: An open-label, acute clinical trial in adults to assess ketone levels, gastrointestinal tolerability, and sleepiness following consumption of (R)-1,3-butanediol (Avela™)
Source: Front Physiol. 2023 Jun 28;14:1195702. doi: 10.3389/fphys.2023.1195702 (PMC10338333; doi:10.3389/fphys.2023.1195702)
Supplement: Supplementary file 1 [file Table1.DOCX]

Supplementary Material

An Open-label, Acute Clinical Trial in Adults to Assess Ketone Production, Gastrointestinal Tolerability, and Sleepiness Following Consumption of (*R*)-1,3-Butanediol (Avela™)

James Lowder, Shafagh Fallah, Carolina Venditti, Kathy Musa-Veloso and Vassili Kotlov

*** Correspondence:** Vassili Kotlov: vassili@impactsciencealliance.org

# Supplementary Data

The following supporting information can be downloaded at: www.mdpi.com/xxx/s1, Table S1 mVAS GI Symptoms Tool; Table S2 Baseline-Adjusted Scores For Belching, Dizziness, and Nausea Symptoms Among those Experiencing Symptoms.

# Supplementary Tables

| Supplementary Table 1: mVAS GI Symptoms Tool | | | | | | | | | | | |
| --- | --- | --- | --- | --- | --- | --- | --- | --- | --- | --- | --- |
|  | **None** | **Mild Symptoms** | | | | **Moderate Symptoms** | | | | | **Severe** |
|  | **0** | **1** | **2** | **3** | **4** | **5** | **6** | **7** | **8** | **9** | **10** |
| **Upper GI Symptoms** | | | | | | | | | | | |
| Belching |  |  |  |  |  |  |  |  |  |  |  |
| Heartburn |  |  |  |  |  |  |  |  |  |  |  |
| Bloating (stomach fullness) |  |  |  |  |  |  |  |  |  |  |  |
| Stomach pain |  |  |  |  |  |  |  |  |  |  |  |
| Urge to regurgitate |  |  |  |  |  |  |  |  |  |  |  |
| Regurgitation |  |  |  |  |  |  |  |  |  |  |  |
| Projectile vomiting |  |  |  |  |  |  |  |  |  |  |  |
| **Lower GI Symptoms** | | | | | | | | | | | |
| Flatulence |  |  |  |  |  |  |  |  |  |  |  |
| Lower abdominal bloating (abdominal pressure) |  |  |  |  |  |  |  |  |  |  |  |
| Urge to defecate |  |  |  |  |  |  |  |  |  |  |  |
| Left intestinal pain |  |  |  |  |  |  |  |  |  |  |  |
| Right intestinal pain |  |  |  |  |  |  |  |  |  |  |  |
| **Defecation** | | | | | | | | | | | |
| Normal consistency |  |  |  |  |  |  |  |  |  |  |  |
| Abnormal loose stools consistency |  |  |  |  |  |  |  |  |  |  |  |
| Diarrhea |  |  |  |  |  |  |  |  |  |  |  |
| Loose stools |  |  |  |  |  |  |  |  |  |  |  |
| **Other GI Symptoms** | | | | | | | | | | | |
| Nausea |  |  |  |  |  |  |  |  |  |  |  |
| Dizziness |  |  |  |  |  |  |  |  |  |  |  |
| Stitch (acute transient) |  |  |  |  |  |  |  |  |  |  |  |

GI: gastrointestinal; mVAS: modified visual analogue scale

In the study by Gaskell et al. (2019), GI symptom severity was rated as none (0), mild (1 to 4), severe (5 to 9), or extremely severe (10), based on the need for cessation of exercise that was being undertaken as part of the study; also, regurgitation and projectile vomiting, as well as all GI symptoms captured under “defecation,” were rated dichotomously, either as absent (0) or severe enough to cause cessation of the prescribed exercise (10). In the study reported herein, GI symptom severity was rated as none (0), mild (1 to 4), moderate (5 to 9), or severe (10), based on the need for cessation of typical movements and actions (e.g., standing, walking, sitting) associated with everyday living; also, because participants were not engaging in prescribed exercise, they were asked to rate the severity of each GI symptom from 0 to 10.

| Supplementary Table 2: Baseline-adjusted Scores for Belching, Dizziness, and Nausea Symptoms Among Those Experiencing Symptoms | | | | | | | | |
| --- | --- | --- | --- | --- | --- | --- | --- | --- |
| **Subject ID** | **Symptom** | **30 min-BL** | **60 min-BL** | **90 min-BL** | **120 min-BL** | **180 min-BL** | **240 min-BL** | **300 min-BL** |
| 5 | Belching | 1 | 2 | 1 | 1 | 1 | 1 | 0 |
| 10 | Dizziness | 0 | 2 | 1 | 0 | 0 | 0 | 0 |
| 15 | Belching | 0 | 0 | 0 | 0 | 1 | 0 | 0 |
| 20 | Belching | 1 | -1 | -1 | -1 | -1 | -1 | -1 |
|  | Dizziness | -2 | -2 | -2 | 3 | -1 | -1 | -2 |
| 23 | Nausea | 1 | 0 | 0 | 0 | 0 | 0 | 0 |
| 24 | Dizziness | 0 | 1 | 0 | 1 | 1 | 1 | 1 |
| 25 | Dizziness | 0 | 0 | 0 | 0 | 1 | 0 | 0 |
| 33 | Belching | 1 | 1 | 0 | 0 | 1 | 0 | 0 |
|  | Dizziness | 0 | 1 | 6 | 8 | 7 | 8 | 2 |
|  | Nausea | 0 | 5 | 6 | 8 | 9 | 9 | 2 |
| 34 | Belching | 1 | 0 | 0 | 0 | 0 | 0 | 0 |
| 39 | Nausea | 1 | 0 | 0 | 0 | 0 | 0 | 0 |
|  | Dizziness | 0 | 0 | 1 | 1 | 0 | 0 | 0 |
| 48 | Dizziness | 0 | 1 | 1 | 0 | 0 | 0 | 0 |
|  | Nausea | 0 | 0 | 1 | 0 | 0 | 0 | 0 |
| 49 | Dizziness | 0 | 0 | 0 | 1 | 0 | 0 | 0 |
| 51 | Belching | 1 | 1 | 1 | 0 | 0 | 0 | 0 |
|  | Dizziness | 1 | 2 | 2 | 2 | 1 | 0 | 0 |
|  | Nausea | 0 | 0 | 0 | 1 | 0 | 0 | 0 |
| 52 | Belching | 0 | 0 | 3 | 3 | 2 | 1 | 0 |
|  | Dizziness | 0 | 0 | 2 | 2 | 1 | 1 | 0 |
| 58 | Belching | 2 | 0 | 0 | 0 | 0 | 0 | 0 |

BL: baseline; ID: identification; min: minutes

A total of 5, 8 and 10 subjects experienced symptoms of nausea, belching and dizziness, respectively. The subjects’ baseline-adjusted scores for each symptom are listed for 30 to 300 minutes. Subjects experiencing combinations of nausea, dizziness, and/or belching can be identified using the Subject ID. Subject 33, who experienced moderate nausea and dizziness, was diagnosed with COVID-19 the morning after the study day. For all other participants, belching, nausea, and dizziness were reported as “mild”.
